# Evidence for the Involvement of p38 MAPK Activation in Barnacle Larval Settlement

**DOI:** 10.1371/journal.pone.0047195

**Published:** 2012-10-24

**Authors:** Li-Sheng He, Ying Xu, Kiyotaka Matsumura, Yu Zhang, Gen Zhang, Shu-Hua Qi, Pei-Yuan Qian

**Affiliations:** 1 KAUST Global Collaborative Research Program, Division of Life Science, Hong Kong University of Science and Technology, Clear Water Bay, Kowloon, Hong Kong SAR, China; 2 South China Sea Institute of Oceanography, Chinese Academy of Science, Guangzhou, China; University of Texas, United States of America

## Abstract

The barnacle *Balanus* (* = Amphibalanus*) *amphitrite* is a major marine fouling animal. Understanding the molecular mechanism of larval settlement in this species is critical for anti-fouling research. In this study, we cloned one isoform of p38 MAPK (Bar-p38 MAPK) from this species, which shares the significant characteristic of containing a TGY motif with other species such as yeast, *Drosophila* and humans. The activation of p38 MAPK was detected by an antibody that recognizes the conserved dual phosphorylation sites of TGY. The results showed that phospho-p38 MAPK (pp38 MAPK) was more highly expressed at the cyprid stage, particularly in aged cyprids, in comparison to other stages, including the nauplius and juvenile stages. Immunostaining showed that Bar-p38 MAPK and pp38 MAPK were mainly located at the cyprid antennules, and especially the third and fourth segments, which are responsible for substratum exploration during settlement. The expression and localization patterns of Bar-p38 MAPK suggest its involvement in larval settlement. This postulation was also supported by the larval settlement bioassay with the p38 MAPK inhibitor SB203580. Behavioral analysis by live imaging revealed that the larvae were still capable of exploring the surface of the substratum after SB203580 treatment. This shows that the effect of p38 MAPK on larval settlement might be by regulating the secretion of permanent proteinaceous substances. Furthermore, the level of pp38 MAPK dramatically decreased after full settlement, suggesting that Bar-p38 MAPK maybe plays a role in larval settlement rather than metamorphosis. Finally, we found that Bar-p38 MAPK was highly activated when larvae confronted extracts of adult barnacle containing settlement cues, whereas larvae pre-treated with SB203580 failed to respond to the crude adult extracts.

## Introduction

**Figure 1:**
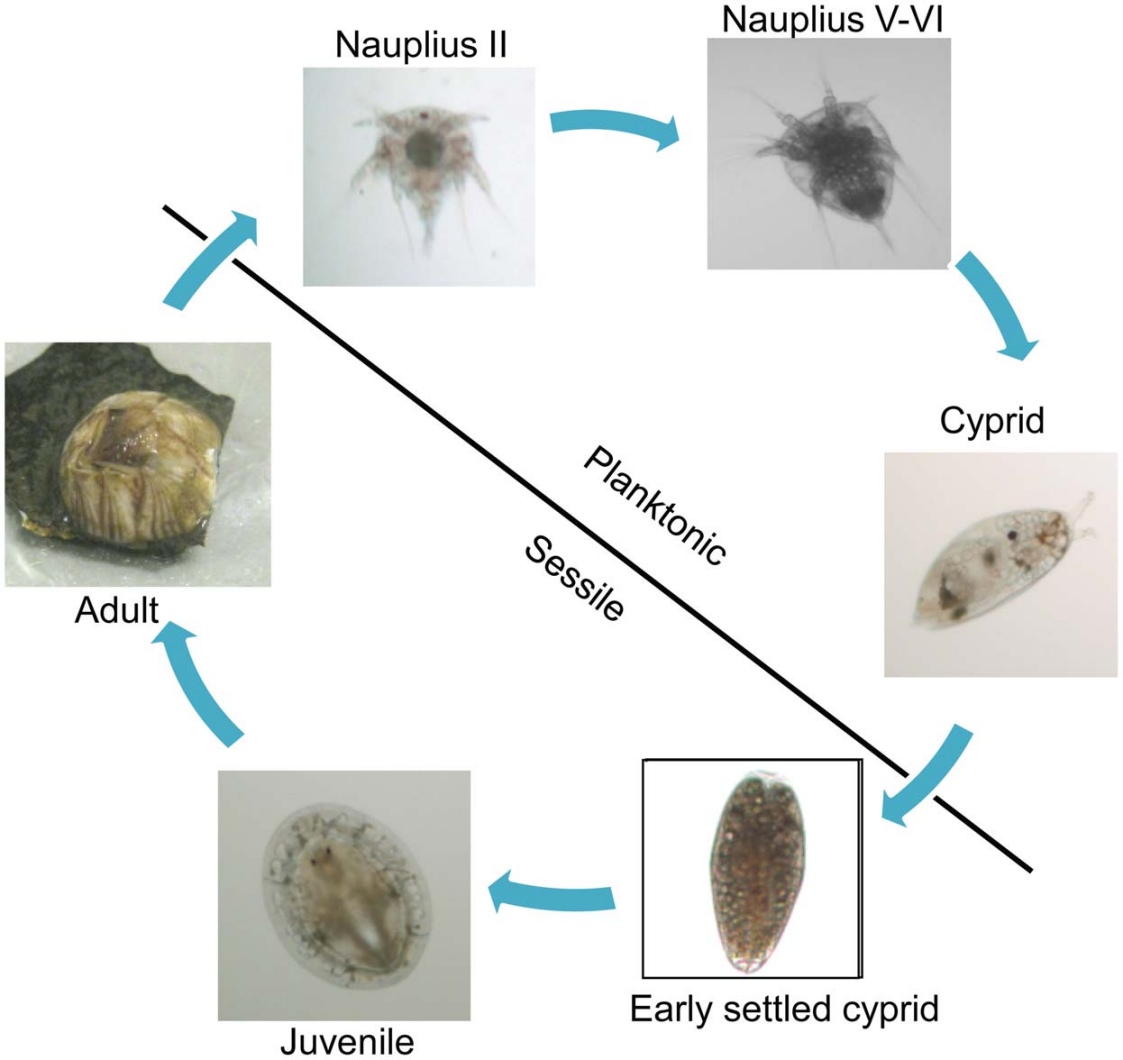
Lifecycle of *B. amphitrite*. Barnacle life is divided into a planktonic phase and a sessile phase. The planktonic phase consists of six nauplius stages and a non-feeding cyprid stage. A cyprid settles to a substratum and then metamorphoses into a juvenile.

It is well known that the barnacle *Balanus amphitrite* is a major fouling marine animal and is widespread throughout the world. The life cycle of this barnacle species consists of six nauplius stages and a non-feeding cyprid stage in the planktonic phase (Fig.1). Before transformation from the planktonic to the sessile phase, cyprids actively explore the surrounding environment with their paired antennules to search for a suitable site for settlement and metamorphosis. Though they also exist in nauplii, the antennules have been highly modified as attachment organs at the cyprid stage. Cyprids detect and examine the substrata for their chemical and physical natures with their antennules that are enriched with neuronal fibers. They then respond and decide whether to settle or not. During this exploration, cyprids temporarily attach to the surface by secreting temporary proteinaceous substances. Once the location is determined, cyprids permanently attach to the surface and then metamorphose into juveniles. Whether larvae can properly settle or not is important for the survival of both adults and subsequent generations [1]. Cyprids thus play a critical role in barnacle development.

Settlement-inducing protein complex (SIPC) has been identified as a pheromone that induces larval settlement [2]. SIPC is a highly glycosylated protein that was isolated from barnacle adult extracts [2]. SIPC is localized not only at the antennules but also in the footprints, which cyprids deposit on the substratum during settlement site exploration [3]–[5]. However, little is known about how SIPC induces larval settlement. Clare and coworkers (1995) demonstrated that adenylate cyclase activator increased the cyprid settlement rate and adenylate cyclase inhibitor prevented cyprid settlement, suggesting that altered endogenous cAMP level affects cyprid settlement. Whereas, radioimmunoassay results have indicated that cAMP level does not change when cyprids are induced to settle using crude adult extracts [6].

Mitogen activated protein kinase (MAPK) signaling transduction pathway displays critical roles in many cellular events, from cell proliferation to apoptosis, by responding to a wide variety of extracellular stimuli. Three classic groups of this pathway have been defined in eukaryotic systems: the extracellular signal-regulated kinase (ERK), C-Jun N-terminal kinase (JNK) and p38 pathways [7]–[9]. The core unit of the MAPK pathway is composed of three enzymes: MAPK, MAPK kinase (MAP2K) and MAPKK kinase (MAP3K). The three-kinase cascade module is activated by a series of phosphorylations. MAPK, which is a serine/threonine-specific kinase, is activated through dual phosphorylations at threonine and tyrosine residues in a conserved loop by MAP2K, which is activated by MAP3K via phosphorylation at serine and/or threonine residues [10].

Recently, Wang and Qian cloned an isoform of p38 from the polychaete *Hydroides elegans*, which shares about a 56% identity and 67% similarity to human p38 alpha. RT-PCR analysis showed that p38 MAPK was highly expressed in competent larvae but not in precompetent larvae. They also showed that SB203580, a specific p38 kinase inhibitor, inhibited the biofilm-induced larval settlement of *H. elegans*. All of these indicated the involvement of p38 MAPK in larval settlement of *H. elegans* [11]. Therefore, there is a large possibility of the involvement of p38 MAPK in barnacle larval settlement. If this is true, it is possible to say that p38 MAPK affects larval settlement by regulating the secretion of proteinaceous substances.

## Results

### Characterization of Bar-p38 MAPK

**Figure 2:**
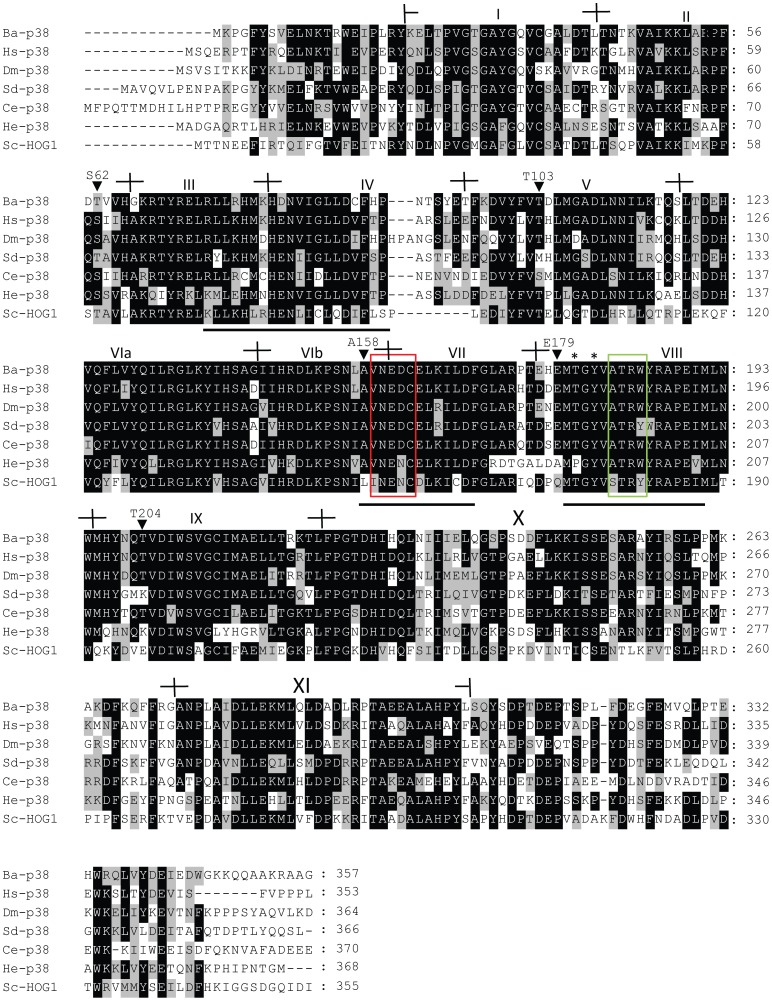
Alignment of the barnacle p38 MAPK sequence with that of other species. *Balanus amphitrite* p38 (Ba-p38, JQ277477) was aligned with human p38α (Hs-p38, Q16539.3), *Drosophila melanogaster* p38α (Dm-p38, NP_477163.1), *Suberites domuncula* p38 (Sd-p38, CAC80141.1), *Caenorhabditis elegans* p38 (Ce-p38, AAB00664.1), *Hydroides elegans* p38 (He-p38, ADC54265.1) and *Saccharomyces cerevisiae* HOG1 (Sc-HOG1, AAA34680.1). The positions of the subdomains I-XI are indicated. The signature sequences are underlined and diagnostic amino acids are indicated by black arrowheads. Kinase interaction motif and substrate binding sites are shown by red and green colored squares, respectively. Conserved dual phosphorylatable residues are indicated with asterisks.

Based on the partial sequence from the barnacle transcriptome database and sequences of the 3′ and 5′ RACE products, the full-length coding region of p38 MAPK was obtained from the barnacle cDNA and was named Bar-p38 MAPK, with 363 deduced amino acids. It has been deposited in GenBank (JQ277477). The alignment of Bar-p38 MAPK with those from other species is shown in Figure 2, with a high homology demonstrated. Two signature sequences – LL[KR]X[LIVM]XHEN[LIVM]IXLLDVF[TS]P and AVNEDCEL[KR][LIVM]LDF – which are distinct from other subfamilies [12], were located at subdomains III-IV and VIb-VII, respectively. Yeast HOG1, which belongs to the YSAPK subfamily, did not share the signature sequences. A global signature sequence [LIVM][TS]XX[LIVM]XT[KR][WY]YRXPX[LIVM][LIVM] [12] that is common to all MAPKs but different from other eukaryotic protein kinases was also found (Fig. 2). Among the three signature sequences, two were perfectly conserved in Bar-p38 MAPK but the first was not. Conserved dual phosphorylation motif TGY for p38 MAPK was also present within the activation loop in Bar-p38 MAPK, suggesting that Bar-p38 MAPK may be activated by MAP2K and share the same regulatory pathway that is conserved in other species. Another important residue, T103 in the ATP docking site, was also conserved in Bar-p38 MAPK (Fig. 2). This residue may interact with the 4-phenyl ring of a highly specific pyridinyl-imidazole inhibitor of human p38α MAPK [13]. This indicates that Bar-p38 MAPK is sensitive to this class of inhibitor, which includes SB203580. Four diagnostic residues consisting of S62, A158, E179, and T204 in *Cyprinus carpio* p38 MAPK have been shown to be particular to the SAPK2 subfamily [12]. All of these residues were conserved in Bar-p38 MAPK except for S62, which was replaced by threonine (Fig. 2).

### Phylogenetic Relationship of Bar-p38 MAPK with Other Species

An overview of the complete sequences indicates that Bar-p38 MAPK shares a high percentage of identity and similarity at the amino acid level with other members of SAPK2, particularly with members of the phylum Arthropoda (Table S2). According to the alignment results, Bar-p38 MAPK has a 71–75% identity with and an 85–88% similarity to p38 MAPKs of ants, shrimps, mosquitoes, and *Drosophila* (Table S2). A relatively lower percentage of identity and similarity were found when Bar-p38 MAPK was compared to p38 MAPKs of humans and mice. Bar-p38 MAPK has the lowest percentage of identity and similarity, about 57% and 75%, respectively, with p38 MAPK of the polychaete *Hydroides elegans* among the species in this study.

**Figure 3:**
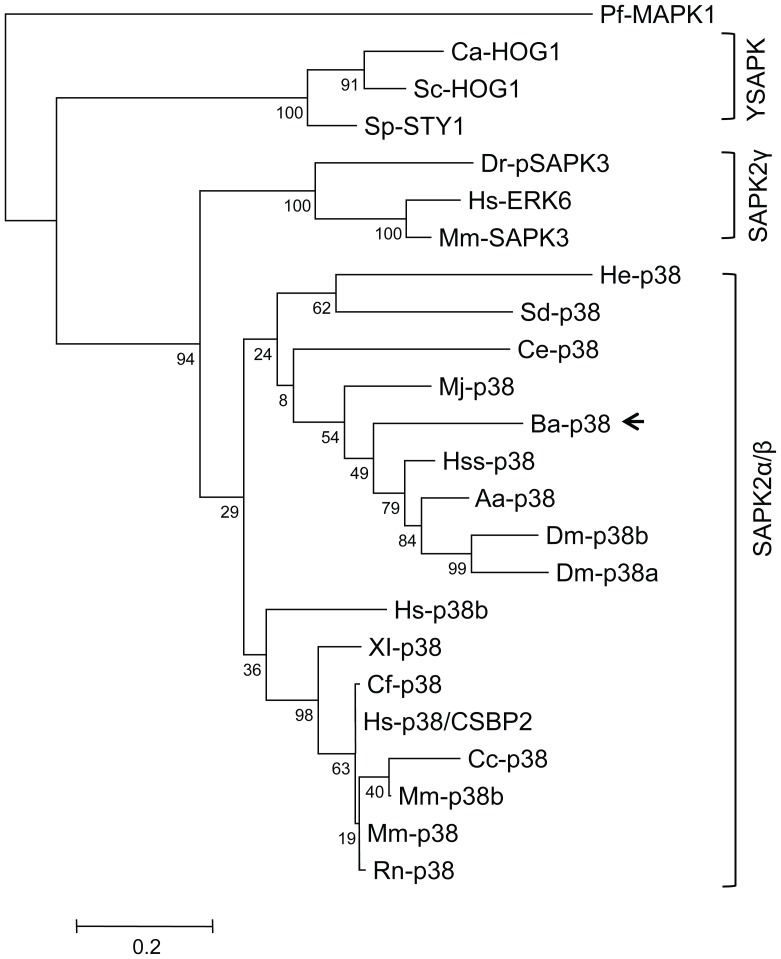
Phylogenetic analysis of MAPKs from the SAPK2 and YSAPK subfamilies. Completed amino acid sequences from alignment plus the following sequences were used. Mm-p38 (mouse, AAA20888.1), Mm-p38b (mouse, BAA19741.1), Rn-p38 (rat, AAC71059.1), Cf-p38 (dog, AAC36131.1), Dm-p38b (*D. melanogaster*, AAC39032.1), Hs-ERK6 (human, CAA55984.1), Mm-SAPK3 (mouse, CAA73850.1), Dr-pSAPK3 (zebrafish, CAA75355.1), Ca-HOG1 (yeast, CAA62214.1), Cc-p38 (*C. carpio*, BAA11881.1 ), Sp-STY1 (yeast, CAA61537.1), Hs-p38b (human, Q15759.2), Hss-p38 (*H. saltator*, EFN89763.1), Aa-p38 (*A. aegypti*, XP_001653240.1), Mj-p38 (*M. japonicus*, BAK78916.1) and Pf-MAPK1 (*P. falciparum*, AAC47170.1). The numbers at the note indicate the occurrences of a branch point over 500 replications. The scale bar indicates the phylogenetic distances. Pf-MAPK1 was used as an outgroup protein. SAPK2: stress activated protein kinase 2. YSAPK: yeast stress activated protein kinase. Ba-p38 was indicated by a black arrow.

From the rooted phylogenetic tree, YSAPK subfamilies branched out from the SAPK2 family and formed a separate group (Fig. 3). Two branches – SAPK2α/β and SAPK2γ – existed in the SAPK2 family. Bar-p38 MAPK was most homologous to *Drosophila*, shrimp, mosquito and ant MAPKs, and clustered with them in the phylogenetic tree. Bar-p38 MAPK was much closer to p38 MAPKs of invertebrates than to those of vertebrates (Fig. 3).

### The Activation of Bar-p38 MAPK is Barnacle Lifecycle Dependent

**Figure 4:**
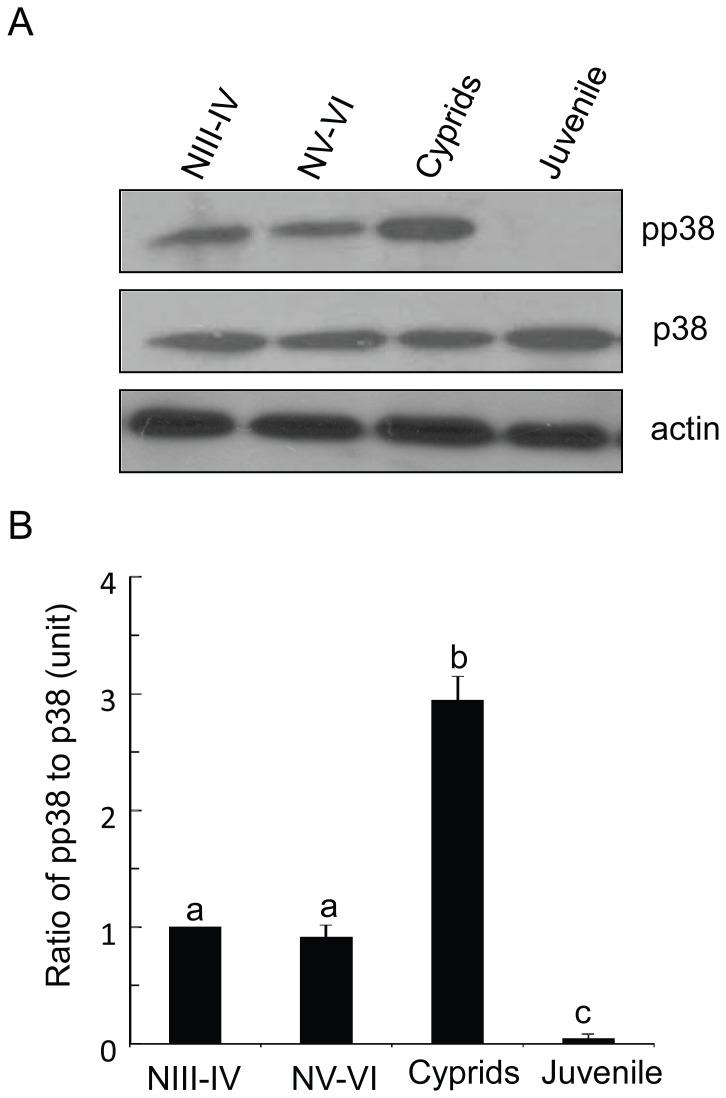
Bar-p38 kinase activation was barnacle lifecycle dependent. (A) pp38 was highly expressed in the cyprid stage. Equal amounts of extracts (60 µg) from NIII-IV, NV-VI, cyprids and juveniles at Day 1 were prepared and blotted with anti-pp38 and anti-total p38 antibodies, respectively. A representative of three experiments is shown. (B) Ratio of pp38 to total p38. The ratio of pp38 to total p38 for NIII-IV represents 1 unit. NIII-IV: nauplii III-IV; NV-VI: nauplii: V-VI. a,b,c, means with different letters are significantly different, P<0.001.

To investigate the role of p38 MAPK in barnacles, protein extracts from different developmental stages, including the nauplius, cyprid, and juvenile stages, were prepared for Western blotting with antibodies against total Bar-p38 MAPK and pp38 MAPK, the active form of this kinase. The results showed that pp38 MAPK was highly expressed in the cyprid stage. The expression level of pp38 MAPK in cyprids was about two- to three-fold higher than that in the nauplius stage and there was a significant difference between these two stages (P<0.001). The expression of pp38 MAPK was extremely low in the juvenile stage, although the expression level of total Bar-p38 MAPK was almost the same in the nauplius, cyprid, and juvenile stages (Fig. 4A,B). The activation of Bar-p38 MAPK, as indicated by the phosphorylation level of this kinase, decreased dramatically and rapidly during the transition from cyprid to juvenile, suggesting the importance of Bar-p38 MAPK in the cyprid stage.

### Bar-p38 MAPK is Localized at Cyprid Antennules

**Figure 5:**
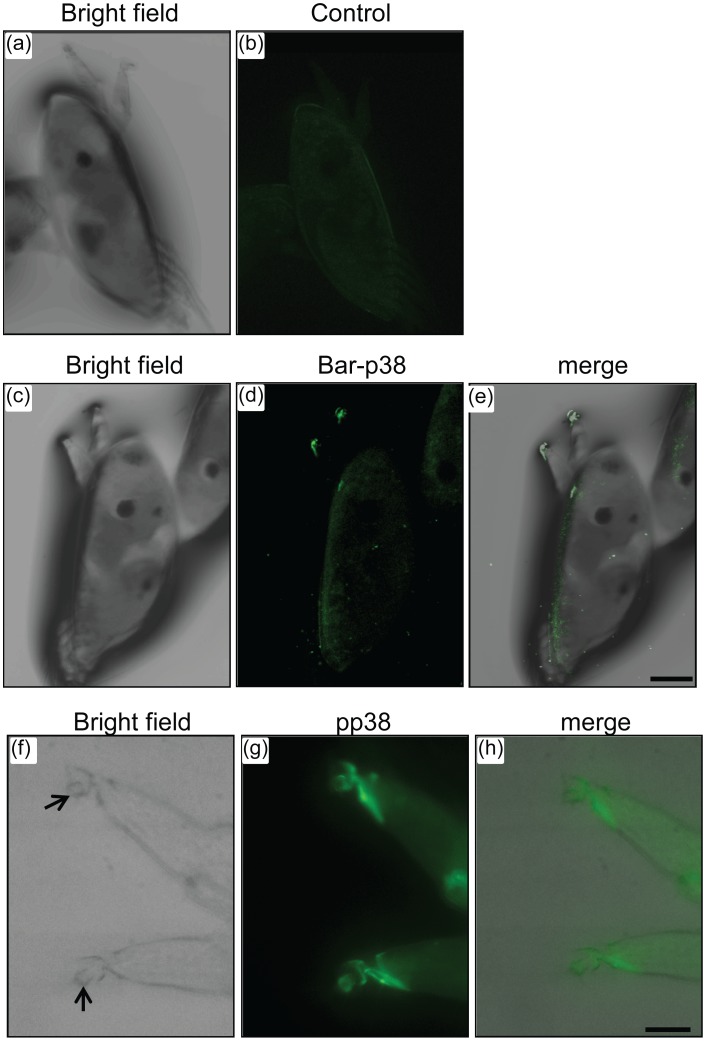
Localization of Bar-p38 in cyprids. Cyprids were fixed with 4% PFA and were then stained with antibody against Bar-p38 and pp38, respectively. Samples stained with only secondary antibody were used as controls. For (a)-(e), 10X objective was used, scale bar: 100 µm; for (f) to (h), 40X objective was used, scale bar: 25 µm. The fourth segments were indicated by black arrows.

To further examine the role of p38 MAPK in the barnacle developmental process, cyprids were immunostained with the antibody against Bar-p38 MAPK. The results showed that Bar-p38 MAPK was highly expressed at the barnacle antennules, particularly at the third and fourth segments of the antennules (Fig. 5). The control image, in which only secondary antibody was used, showed no significant localization pattern (Fig. 5). Higher magnification images were observed to further confirm the localization of pp38 MAPK, the active form of p38 MAPK, on the third and fourth segments using a pp38 antibody (Fig. 5).

### Bar-p38 MAPK is Required for the Larval Settlement

**Figure 6:**
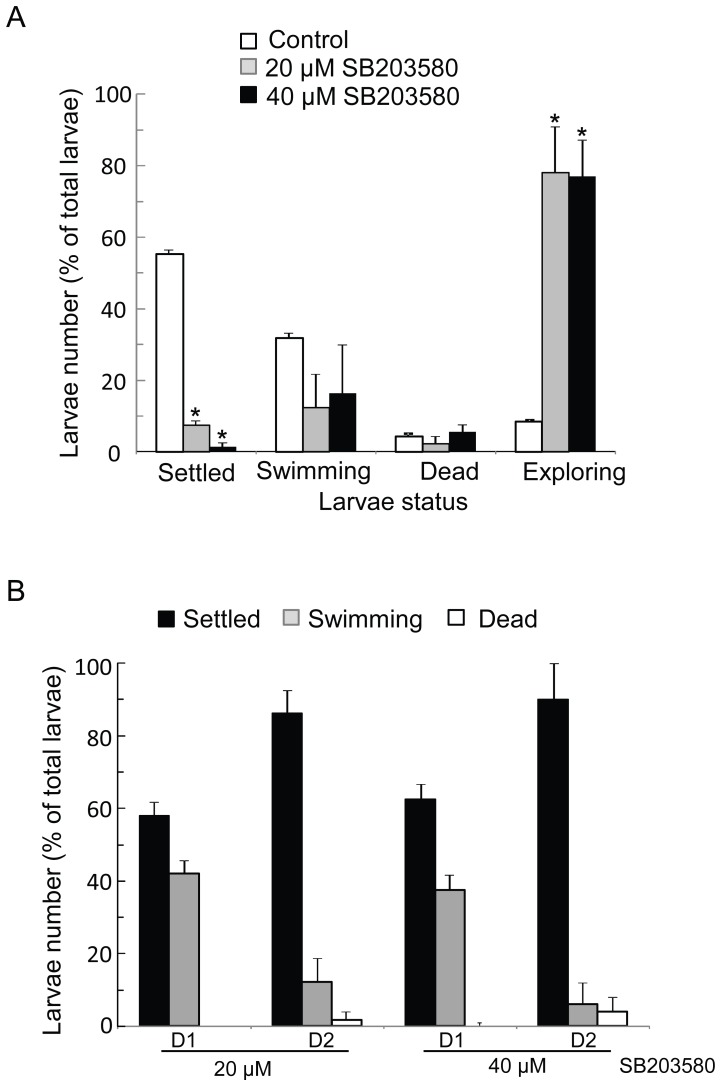
Bar-p38 kinase was required for larval settlement. (A) Cyprids were treated with p38 kinase inhibitor SB203580 at two concentrations, as indicated in the figure, to perform the settlement bio-assay. Settled, swimming, dead and exploring larvae were counted after one day of treatment. *P<0.05 vs. control groups. (B) After one day of treatment, the experimental medium was replaced with FSW and incubated for another two days. Settled, swimming and dead larvae were counted. The data shown is the mean ± S.E. All of the bioassays were performed with three replicates and at least three batches.

SB203580, a specific p38 MAPK inhibitor, was used to examine the role of the kinase in larval settlement [14]. We observed that more than 50% of larvae settled in the control groups after 24 hours of incubation. However, less than 10% and 1% of larvae settled after treatment with 20 µM and 40 µM of SB203580, respectively, displaying a significant reduction in comparison to the control (P<0.001) (Fig. 6A). The numbers of dead larvae were less than 5% and there was no significant difference between the treated and untreated samples (P>0.05). This signifies the low toxicity of this inhibitor to the larvae (Fig. 6A). Interestingly, although most of the larvae in the treatment groups could not settle, they were still able to temporarily attach to the surface of the plate. This phenomenon suggests that treatment with SB203580 may result in metamorphosis impairment following settlement disruption.

Based on the inhibition mechanism of SB203580 on p38 MAPK, which reversibly competes for ATP binding site on p38 MAPK, we performed a recovery settlement bioassay to further examine whether the behavior that we observed was specific to Bar-p38 MAPK. The results showed that about 60% and 80% of larvae settled in 24 and 48 hours after removal of SB203580, respectively, for each concentration treatment (Fig. 6B). Taken together, the results indicate that SB203580, a p38 MAPK specific inhibitor, significantly and reversibly inhibits cyprid settlement in a dose-dependent manner.

### The Activation of Bar-p38 MAPK may be Highly Related to Cyprid Settlement Rather than Metamorphosis

**Figure 7:**
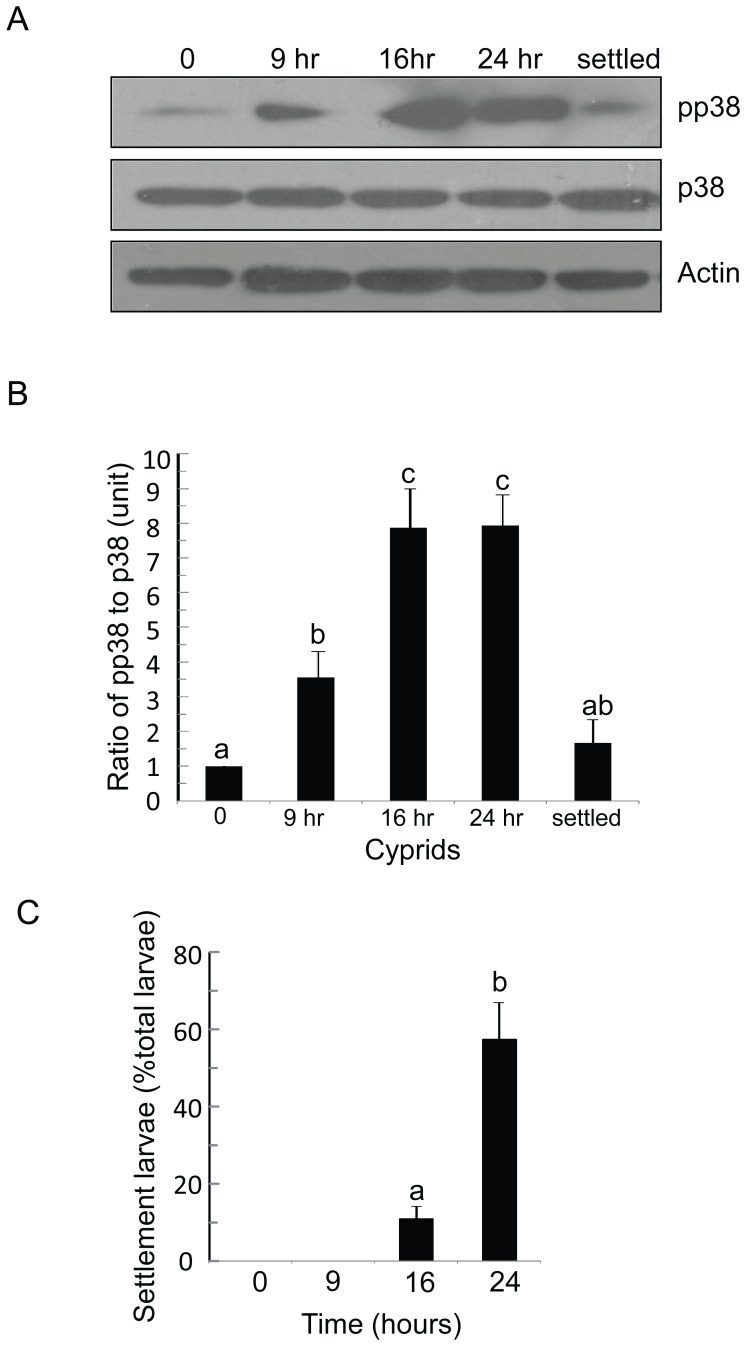
Bar-p38 kinase activation may be highly related to cyprid settlement rather than metamorphosis. ( A) Fresh cyprids (time 0) were placed in polystyrene (PS) containers and collected at different time points, as indicated. Non-settled cyprids from different time points and early settled cyprids were collected and prepared for blotting with antibodies against pp38, p38 and actin, respectively. (B) The ratio of pp38 to total p38 presented as the mean ± S.E. of three replicates. The ratio of pp38 to total p38 for time 0 represents 1 unit. a,b,c, means with different letters are significantly different, P<0.05. (C) Settlement rate at different time points. The data shown is the mean ± S.E. of three replicates. a,b, means with different letters are significantly different, P<0.001. hr: hour. Settled: early settled cyprids.

To further investigate the role of Bar-p38 MAPK in the cyprid settlement process, young cyprids (within 2 hours of molting from nauplius VI to cyprid) were placed in a polystyrene plate and the settled larvae were counted at various time points. Non-settled cyprids and early settled cyprids (before metamorphosis into juveniles) were harvested and proteins were extracted for the detection of the total Bar-p38 MAPK and pp38 MAPK by their particular antibodies. The results indicated that the pp38 MAPK level increased gradually from 0 to 24 hours (Fig. 7A). The expression level ratios of pp38 MAPK to total p38 MAPK at 9 and 16 hours were more than four-fold and six-fold than that at 0 hour, respectively, showing a significant difference between these time points (P<0.05) (Fig. 7B). The larval settlement rate also increased gradually. At 0 and 9 hours, no larvae had settled. About 10% of larvae had settled at 16 hours, and 50% had settled at 24 hours and there was a significant difference between these two time points (P<0.001) (Fig. 7C). Interestingly, pp38 MAPK levels decreased dramatically and rapidly in the early settled cyprids. At this stage, the cyprids have just finished settlement but have not completed metamorphosis. These results suggest the importance of Bar-p38 MAPK in larval settlement rather than metamorphosis.

### p38 MAPK Inhibitor Impairs Adult Extract-induced Larval Settlement

**Figure 8:**
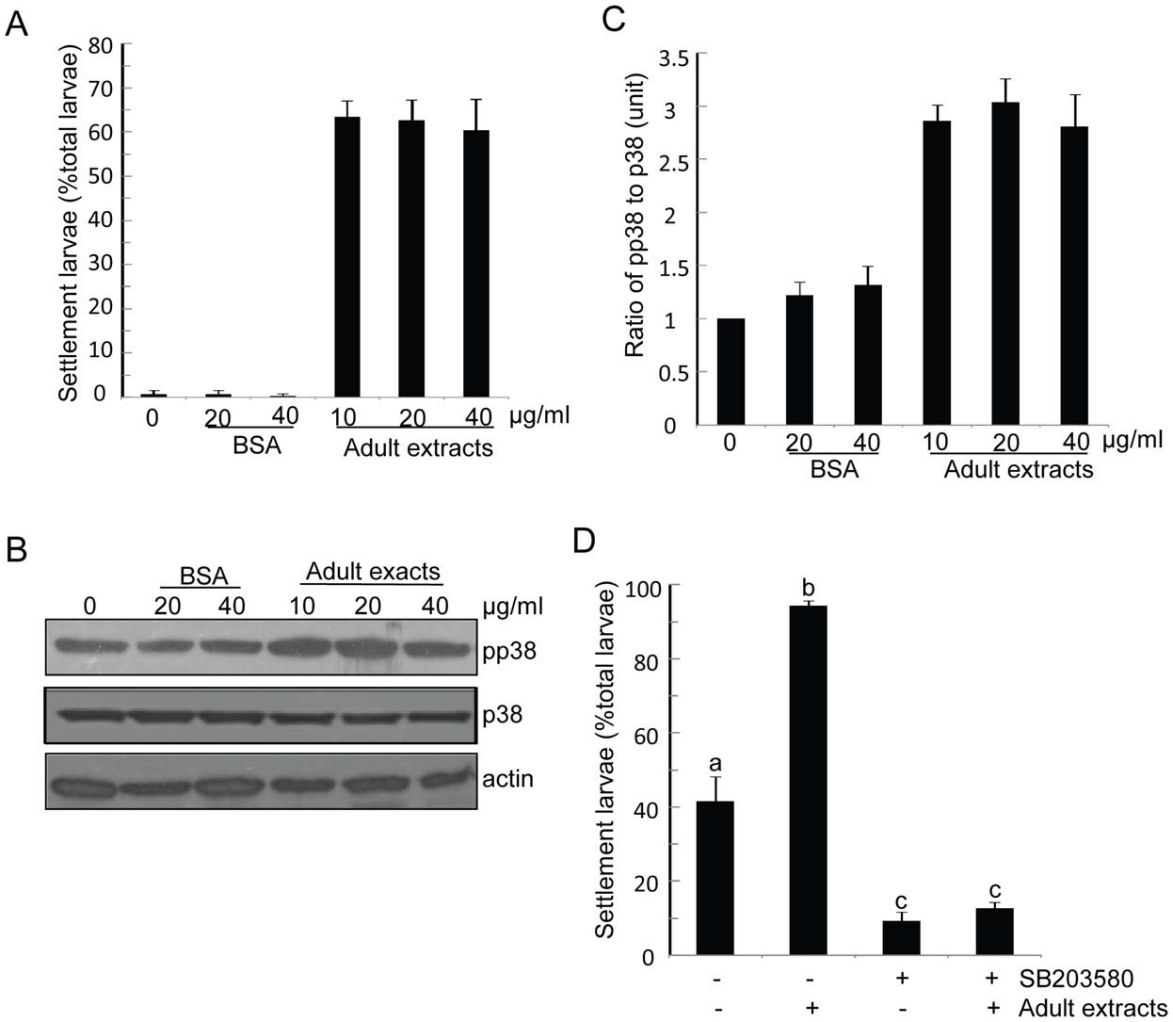
p38 kinase inhibitor impaired inducement of larvae settlement by adult extracts. (A) Fresh cyprids were placed in PS containers and treated with BSA or crude adult extracts. Settled larvae were counted after treatment for 8 hours. (B) Non-settled cyprids were collected after treatment for 8 hours and prepared for blotting with antibodies against actin, p38 and pp38, respectively. (C) The ratio of pp38 to p38 was presented as the mean ± S.E. of three replicates for each batch (at least three batches). For (A) and (C), Two-Way ANOVA was performed. The result indicated that adult extracts significantly affected larval settlement and pp38 level (P<0.05). Different doses used in this assay have no significant difference (P>0.05). (D) Cyprids were treated with only 20 µg ml^−1^ of adult extracts or 20 µM of SB203580, respectively, or pre-treated with 20 µM of SB203580 for 24 hours and then plus 20 µg ml^−1^ of adult extracts (double treatment). Settled cyprids were counted after treatment with adult extracts for 24 hours. a,b,c, means with different letters are significantly different, P<0.001.

Crude extracts of barnacle adults are well known to be a natural inducer of conspecific larval settlement [15]. However, how such extracts induce larval settlement is unclear. In this study, larvae were treated with three doses of adult extracts –10 µg ml^−1^, 20 µg ml^−1^, and 40 µg ml^−1^. Untreated larvae and larvae treated with BSA at 20 and 40 µg ml^−1^ were used as controls. Settled larvae were counted and unsettled larvae were collected for Western blotting. The results showed that more than 60% of the larvae settled after 8 hours of treatment with the adult extracts. There was no significant difference between the different protein doses of the extracts (P>0.05). However, less than 5% of the larvae settled in the controls, which is a significant difference compared to that in the treated larvae with adult extracts (P<0.05) (Fig. 8A). The quantification of pp38 MAPK levels after blotting revealed that pp38 MAPK increased about two-fold after treatment with the adult extracts compared with the controls and there was a significant difference between them (P<0.05) (Fig. 8B,C). These results suggest that adult extract-induced larval settlement was achieved through activation with Bar-p38 MAPK. To further verify this hypothesis, larvae were pre-treated with p38 MAPK inhibitor and then treated with the crude adult extracts. The results showed that the adult extracts lost the ability to induce larval settlement when the larvae had been pre-treated with p38 MAPK inhibitor (Fig. 8D).

## Discussion

We cloned one isoform of p38 MAPK from the barnacle *Balanus amphitrite* that shares a conserved TGY phosphorylation motif with other organisms such as, *Drosophila*, and humans. The bioassay with the p38 MAPK inhibitor indicated the involvement of p38 MAPK in larval settlement. The results also suggested that a natural inducer, SIPC, which is included in crude adult extracts, induces larval settlement via the mediation of the p38 MAPK pathway. These findings give us a new vision in the mechanism of larval settlement.

The cyprid stage plays a critical role in the transition from the planktonic to the sessile phase of barnacles (Fig. 1). However, the molecular mechanism that controls larval settlement remains largely unknown. The dual phosphorylation in the TGY motif was detected with a pp38 MAPK antibody, which indicated the active form of p38 MAPK [16]. In this study, we found that pp38 MAPK was highly expressed in the cyprid stage, especially in aged cyprids that were highly competent to settle (Figs. 4 and 7), suggesting a close relationship between the activation of Bar-p38 MAPK and cyprid settlement. This is consistent with other studies, which have shown that aged cyprids generally have a higher settlement rate than younger cyprids [17], [18]. Given the importance of cyprid antennules for larval settlement during exploration [19], the localizations of Bar-p38 MAPK and pp38 MAPK, the active form of p38 MAPK, at the antennules also indicates a linkage between Bar-p38 MAPK and larval settlement (Fig. 5). This hypothesis was further supported by a settlement bioassay using the p38 MAPK inhibitor SB203580 (Fig. 6).

The barnacle changing from planktonic to sessile phase is very complicated, including settlement and metamorphosis processes. However, it is difficult to separate these two processes clearly (Fig.1). After SB203580 treatment, most of the cyprids could not settle and metamorphose but were still capable of exploring (temporary attachment) (Fig. 7 and Movie S1). From this result, we can deduce that the larval settlement is somehow affected by SB203580. There are two possible explanations for the defect in metamorphosis. One is that SB203580 affects metamorphosis directly. Metamorphosis is a very close following step after settlement. During the normal physiological process of settlement and metamorphosis, directly after or towards the end of settlement, there should be some internal signals to trigger the subsequent metamorphosis. So the second possibility is the indirect effect of p38 MAPK on cyprid metamorphosis. This means that there was no metamorphosis due to the lack of settlement. In our experiments, one result in Fig.7A showed that pp38 level decreased after settlement, which supports the second theory. Therefore, the defective metamorphosis is perhaps a secondary effect following settlement impairment due to SB203580 treatment.

SIPC is a well defined pheromone that induces larval settlement in barnacles [2]. However, its perception and signal transduction mechanisms have not been clarified. Although an altered cAMP level may change the larval settlement rate, endogenous cAMP level in cyprids does not change after treatment with crude adult extracts [6]. In this study, we found that pp38 MAPK obviously increased after treatment with adult extracts for 3 hours in comparison to the controls (data not shown). After 8 hours of treatment, the settlement rate was about 80%, whereas in the control group it was less than 5%. The pp38 MAPK level was about two times higher than that in the controls, suggesting that adult extracts induce larval settlement through the activation of Bar-p38 MAPK. We used adult extracts instead of purified SIPC to induce larval settlement. Although adult extracts acting as a common natural settlement inducer have been used in many experiments [6], [15], [20], the increase in pp38 MAPK due to exposure to adult extracts may not be mediated by SIPC, or at least not by SIPC alone. To further investigate whether SIPC induces larval settlement through the p38 MAPK pathway, larvae were pre-treated with SB203580 to block phosphorylation events catalyzed by Bar-p38 MAPK and were then exposed to adult extracts. The results showed that larvae pre-treated with SB203580 failed to respond to the adult extracts. This suggests that the p38 MAPK pathway is downstream of the larval settlement signaling transduction pathway stimulated by SIPC.

Three types of MAPK pathways – p38 MAPK, ERK, and JNK – are conserved from yeast to humans [16]. We also examined phosphorylated ERK (pERK) and phosphorylated JNK (pJNK) using antibodies against pJNK or pERK on dual phosphorylated residues in a TXY motif. Equal amount of extracts from nauplii, cyprids and juveniles were prepared and blotted against pp38, pERK and pJNK to understand the expression patterns of these three active forms in different developmental stages. Similar levels of pERK were detected in the nauplius and cyprid stages, and were much higher than that in the juvenile stage. The protein level of pJNK was similar in the cyprid and juvenile stages, but a little higher than that in the nauplius stage. In contrast, pp38 MAPK was more highly expressed in the cyprid stage than that in the nauplius and juvenile stages. The different expression patterns of the phospho-MAPKs indicate their distinct functions during barnacle development (Fig. S1).

Our data clearly show the involvement of bar-p38 MAPK in barnacle larval settlement. However, how Bar-p38 MAPK regulates larval settlement remains unknown. Immunostaining showed that Bar-p38 MAPK is mainly localized at the antennules, which are important sensory organs responsible for settlement site exploration during larval settlement. Although they also exist in nauplii, the antennules are highly modified as attachment organs at the cyprid stage [19]. Cyprid antennules are composed of four segments. The first and second segments are responsible for antennule movement during bi-pedal walking on a surface [21]. The third segment is covered with cuticular villi, which increase the area between the surface and the antennules during walking. Numerous duct terminals open to the disk of the third segment, where proteinaceous substances are secreted for adhesion. The fourth segment stretches out from outside of the third segment and is filled with neuronal fibers [21]. A liquid proteinaceous substance from the cement gland is thought to play a critical role during temporary and permanent attachment. For temporary adhesion, the adhesive originates from specialized hypodermal glands in the second segment. In contrast, the permanent adhesive is directly excreted from paired cement glands within the cyprid body [22]. This adhesive is delivered through the antennular cement duct and deposited in a globular disc that is close to the surface of the antennular adhesive disk in a settled cyprid [23], [24]. These indicate the different regulation pathways of temporary and permanent attachment. In this study, SB203580 treatment disrupted larval permanent adhesion but not temporary adhesion. The larvae still had the ability to explore on the substratum surface after SB203580 treatment. We further found that Bar-p38 MAPK was localized at the third and fourth segments of the antennules. All of these findings suggest that Bar-p38 MAPK may regulate larval settlement by controlling the secretion of permanent proteinaceous substances. The composition of the temporary and permanent adhesives should be different based on their distinct functions. Therefore, to further prove the role of p38 in this process, both of cement proteins could be examined after the treatment of SB203580.

The core unit in the MAPK pathway is made up of three main members: MAPK, MAP2K and MAP3K. In the p38 MAPK pathway, MAPKK3 and MAPKK6 are specific to p38 MAPK phosphorylation and activation [25]. Bar-p38 MAPK shares all of the common characteristics with other members of the SAPK2 family, including distinct sequences, diagnostic residues and a dual phosphorylatable TGY motif. The common properties between Bar-p38 MAPK and the p38 MAPKs of other species indicate that they may share similar regulation pathways. In our barnacle transcriptome database [26], we found a partial sequence that is similar to MAPKK3/MAPKK6 (data not shown), which indicates that Bar-p38 MAPK may be regulated by conserved MAPKK3/MAPKK6.

In conclusion, p38 MAPK is involved in larval settlement and the p38 kinase inhibitor SB203580 impaired adult extract-induced larval settlement.

## Materials and Methods

### Ethics Statement

The barnacle of *Balanus amphitrite* is a common species of marine invertebrate. It is a biofouling species and not endangered or protected. Adult barnacles of *Balanus amphitrite* were collected from populations growing on a concrete pole at Pak Sha Wan in Hong Kong (22°21′45′’N, 114°15′35′’E). No specific permits were required for the adult barnacle collection. The dock does not belong to any national parks, protected areas, or privately owned places. The field studies did not involve any endangered or protected species.

### Larval Culture

Larvae were released and cultured in the laboratory according to the procedure of Zhang et al (2010). In brief, the released larvae were maintained at a density of 1 larva ml^−1^ in 0.22 µm filtered seawater (FSW) at 28°C and fed with *Chaetoceros gracilis* [27]. Nauplii became cyprids on day 4 after six molts from nauplius I to cyprid.

### p38 cDNA Isolation

RNA extraction and cDNA synthesis were performed according to the methods of Chen et al (2011). Briefly, the total RNA was extracted by using TRIzol reagent (Invitrogen, USA), and cDNA was then prepared from the total RNA using M-MLV reverse transcriptase (Ambion, USA) with the oligo dT primer [26]. Based on the partial sequence of p38 in the barnacle transcriptome database [26], primers were designed together with rapid amplification of cDNA ends (RACE) reactions to obtain the full-length coding sequence of p38 [11]. The primers that were used to obtain the full-length sequence of p38 are listed in Table S1. The full-length cDNA of p38 was further confirmed by sequencing (BGI Company, China).

### Sequence Comparison

Multiple alignments were performed with the CLUSTAL W version 1.83 program and were graphically presented with the software BioEdit 7.0.0. The rooted phylogenetic tree was constructed with amino acid sequences by the Maximum Likelihood statistical method with the MEGA software (version 5.05). The degree of support for internal branches was further assessed by bootstrap analysis with 500 replications.

### Plasmid Construction, Recombinant Protein Over-expression and Antibody Generation

One fragment derived from the p38 sequence – from a.a.15 to a.a.101– was used to generate the antibody. The fragment was cloned into pGEX4T and pet21b vectors, respectively, and then overexpressed in *E. coli* BL21 (DE3). Recombinant proteins containing GST or His_6_ were isolated according to a previous publication [28] and the manufacture’s protocol using GST-Sepharose (Sigma, USA) or Ni^2+^ -nitrilotriacetic acid beads (Qiagen, USA), respectively. The procedure for the injection and purification of the antibody were performed according to a previous publication [29]. In brief, His_6_ tagged recombinant proteins were used as antigens to immunize New Zealand white rabbit with Freund’s adjuvant (Sigma, USA). Serum was collected after four injections. GST-tagged recombinant protein was used as bait to isolate the antibody.

Antibodies against pp38 (Thr180/Tyr182), phospho-ERK (Thr202/Tyr204), phospho-JNK (Thr183/Tyr185), and anti-rabbit and mouse HRP labeled secondary antibodies were purchased from Cell Signaling Technology (USA). Anti-rabbit Alexafluor488 was purchased from Invitrogen (USA), and anti-actin antibody was from Millipore (USA).

### Protein Extraction and Western Blot Analysis

Larvae from different stages or time points, as indicated in the figures, were collected and then lysed in lysis buffer (25 mM Tris-HCl pH 7.5, 300 mM NaCl, 1% sodium dodecyl sulfate (SDS), 1% Triton X-100, and 1 mM dithiothreitol (DTT) plus protease inhibitors and phosphatase inhibitors (Roche, Germany)). After sonication (Branson Digital Sonicator 250, Danbury, CT), the samples were centrifuged at 20 k×g to pellet the debris and to collect the supernatants. The protein concentration was measured by the Bradford and RC-DC methods (Bio Rad, USA). Crude extracts (60 µg of proteins) were subjected to SDS-PAGE gel and then transferred to a 0.22 µm-PVDF membrane (Millipore, USA) for Western blotting with various antibodies, as indicated in the figures.

For the Western blot quantification, images of anti-total p38 and pp38 were acquired by using a ChemiDoc XRS system (Bio-Rad, USA) and analyzed with the Quantity One software (Bio-Rad, USA).

### Immunofluorescence Imaging

After washing with FSW once, the cyprids were fixed with 4% paraformaldehyde (PFA) in phosphate-buffered saline (PBS) overnight at 4°C. For permeabilization, all of the samples were sonicated in the same buffer five times at an output level of 2 and a cycle level of 2, and then incubated with 0.5% Triton X-100 in PBS for 30 minutes at room temperature. After blocking with 5% bovine serum albumin (BSA) in PBS, the cyprids were subjected to immunostaining with the p38 and pp38 antibodies, respectively. Primary and secondary antibodies were diluted in 5% BSA in PBS buffer with 1∶300 and 1∶1000 dilution, respectively. Fixed cyprids were incubated with primary and secondary antibodies overnight at 4°C. Between these two incubations, washing with PBS was performed 3 times between 15-minute intervals. Cyprids stained only with anti-rabbit secondary antibody served as the controls. Images were observed under a laser scanning confocal microscope (Zeiss, LSM 710, ZEN 2009 software, USA).

### Live Imaging

For live imaging, larvae were treated with 20 µM of p38 kinase inhibitor SB203580 for 24 hours, and an EMCCD camera (SPOT BOOST BT2100; Diagnostic instruments) was used to capture the images of their behavior at one picture every 5 seconds for 5 minutes. TIF image stacks were exported as MOV files using MetaMorph software (Molecular Devices, Sunnyvale, CA). The playback rate was six frames a second.

### Settlement Bioassay

Cyprid larvae (at 2 hours or 1 day after molting from nauplius VI to cyprid) were collected and placed in sterile 24-well or 6-well polystyrene Petri dishes (NUNC, Denmark) to perform the larval settlement assay. Fifteen to 20 cyprids were placed into each well of a 24-well plate (50 to 55 for a 6-well plate), and incubated at 25°C with or without SB203580 (LC Laboratory, Woburn, MA). SB203580 was dissolved in dimethylsulfoxide (DMSO) at 50 mM as a stock solution. One day after the treatment, the larvae were observed under a dissecting microscope.

For the recovery assay, the experimental solution containing p38 kinase inhibitor was removed after one day of treatment. After washing with FSW once, the samples were re-incubated with FSW for another two days. Daily observations were performed under a dissecting microscope.

For the assay in the presence of settlement cues, crude adult extracts were prepared according to the procedure in a previous study [15]. Briefly, the adult barnacles were crushed and homogenized in FSW. The supernatant was collected after centrifugation at 20 k×g. The cyprids were treated with different doses of crude adult extracts or BSA as indicated in Figure 8. Settled cyprids were counted after 8 hours of treatment and unsettled cyprids were collected for Western blotting.

For the inducing settlement assay with p38 kinase inhibitor, the cyprids were pre-treated with 20 µM of SB203580 for 24 hours, and the crude adult extracts were then added at 20 µg ml^−1^ as a final concentration. Settled cyprids were counted after one day. All of the bioassays were performed for at least two batches of larvae with three replicates each.

### Statistics

Statistical analysis was performed by SPSS 16.0 using One-Way ANOVA followed by Tukey’s post hoc test for comparison among multiple groups and using Student’s unpaired two tails *t* tests for comparison between two groups except the indication. For all comparisons, P<0.05 was considered statistically significant.

## Supporting Information

Figure S1 **Temporal expression patterns of three MAPKs in** ***B. amphitrite*** **.** (A) Equal amounts of extracts (60 µg) from nauplii III-IV, cyprids and juveniles were prepared and blotted with anti pp38, pERK and pJNK antibodies, respectively. (B) The ratio of pp38, pERK and pJNK to actin from nauplii III-IV, cyprids and juveniles are presented as the mean ± S.E. of three replicates. For pp38 and pERK, the ratio from juveniles represents 1 unit; for pJNK, the ratio from nauplii III-IV represents 1 unit. P values are indicated.
(TIF)

Table S1 **Primers used for isolation full-length bar-p38.**
(TIF)

Table S2 **Identity and similarity between bar-p38 and other SAPK2.**
(TIF)

Movie S1 **Cyprid larvae were treated with 20 µM of SB203580 for 24 hours.** Images were recorded at one picture every 5 seconds for 5 minutes with a camera attached to a microscope and stacked into a movie with the rate of six frames a second. The live images showed that cyprid larvae could still move and explore on the surface but lost the ability of permanent attachment and metamorphosis after SB203580 treatment, indicating the involvement of p38 MAPK in the larval permanent attachment.
(ZIP)
