# Supplementary figures and images for: Evidence for the Involvement of p38 MAPK Activation in Barnacle Larval Settlement
Source: PLoS One. 2012 Oct 24;7(10):e47195. doi: 10.1371/journal.pone.0047195 (PMC3480373; doi:10.1371/journal.pone.0047195)

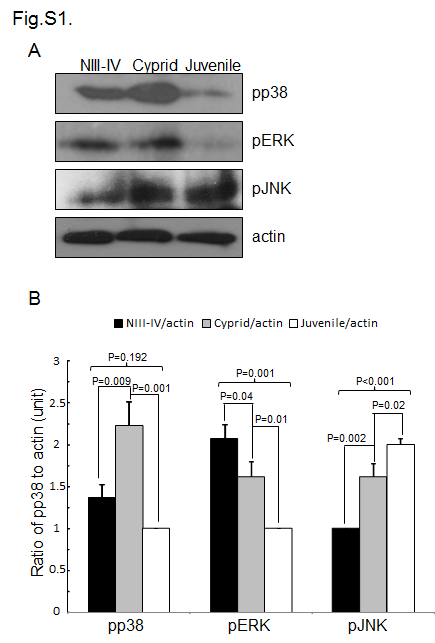

Supplement: Figure S1 — Temporal expression patterns of three MAPKs in B. amphitrite . (A) Equal amounts of extracts (60 µg) from nauplii III-IV, cyprids and juveniles were prepared and blotted with anti pp38, pERK and pJNK antibodies, respectively. (B) The ratio of pp38, pERK and pJNK to actin from nauplii III-IV, cyprids and juveniles are presented as the mean ± S.E. of three replicates. For pp38 and pERK, the ratio from juveniles represents 1 unit; for pJNK, the ratio from nauplii III-IV represents 1 unit. P values are indicated. (TIF) [file pone.0047195.s001.tif]

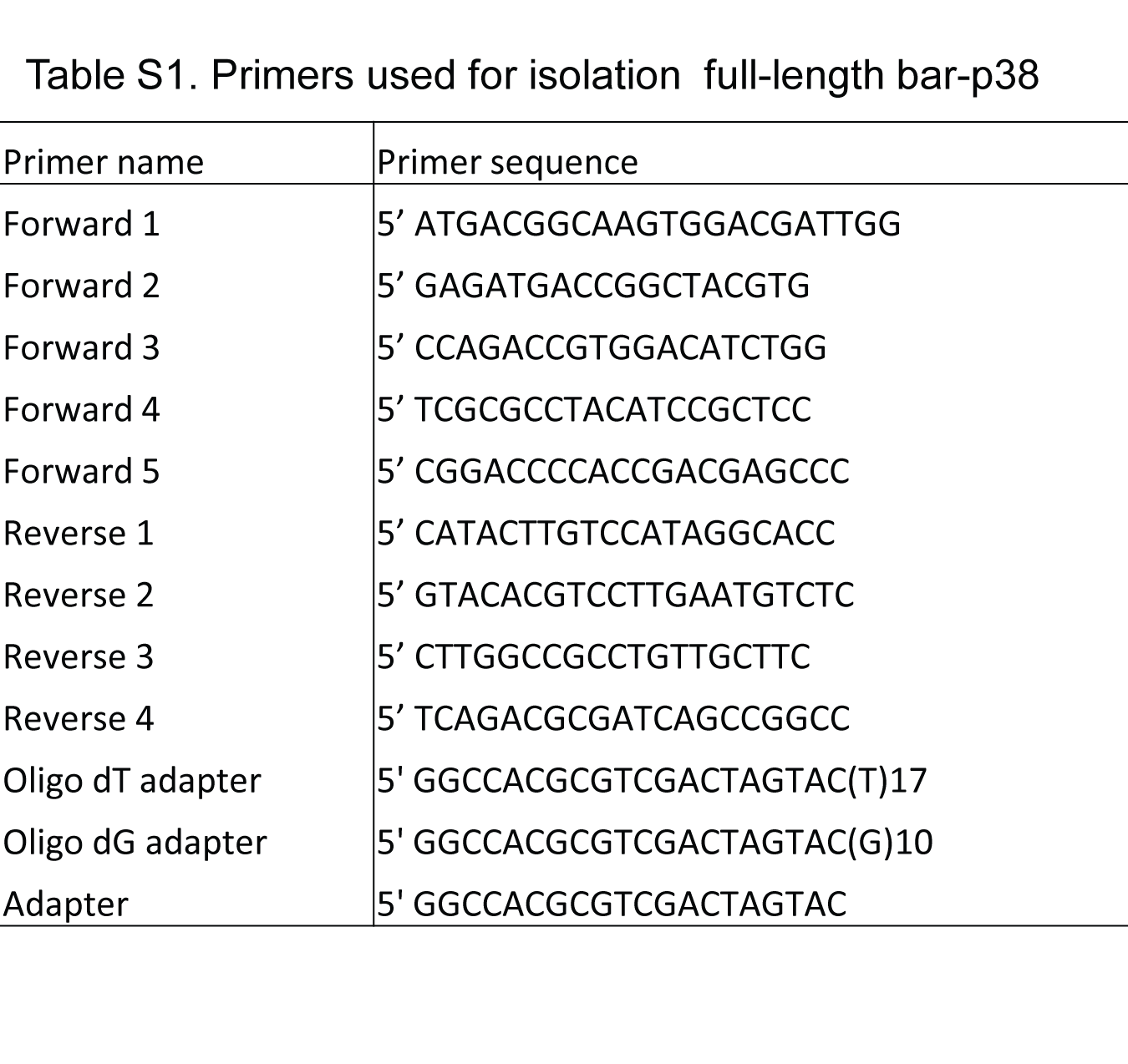

Supplement: Table S1 — Primers used for isolation full-length bar-p38. (TIF) [file pone.0047195.s002.tif]

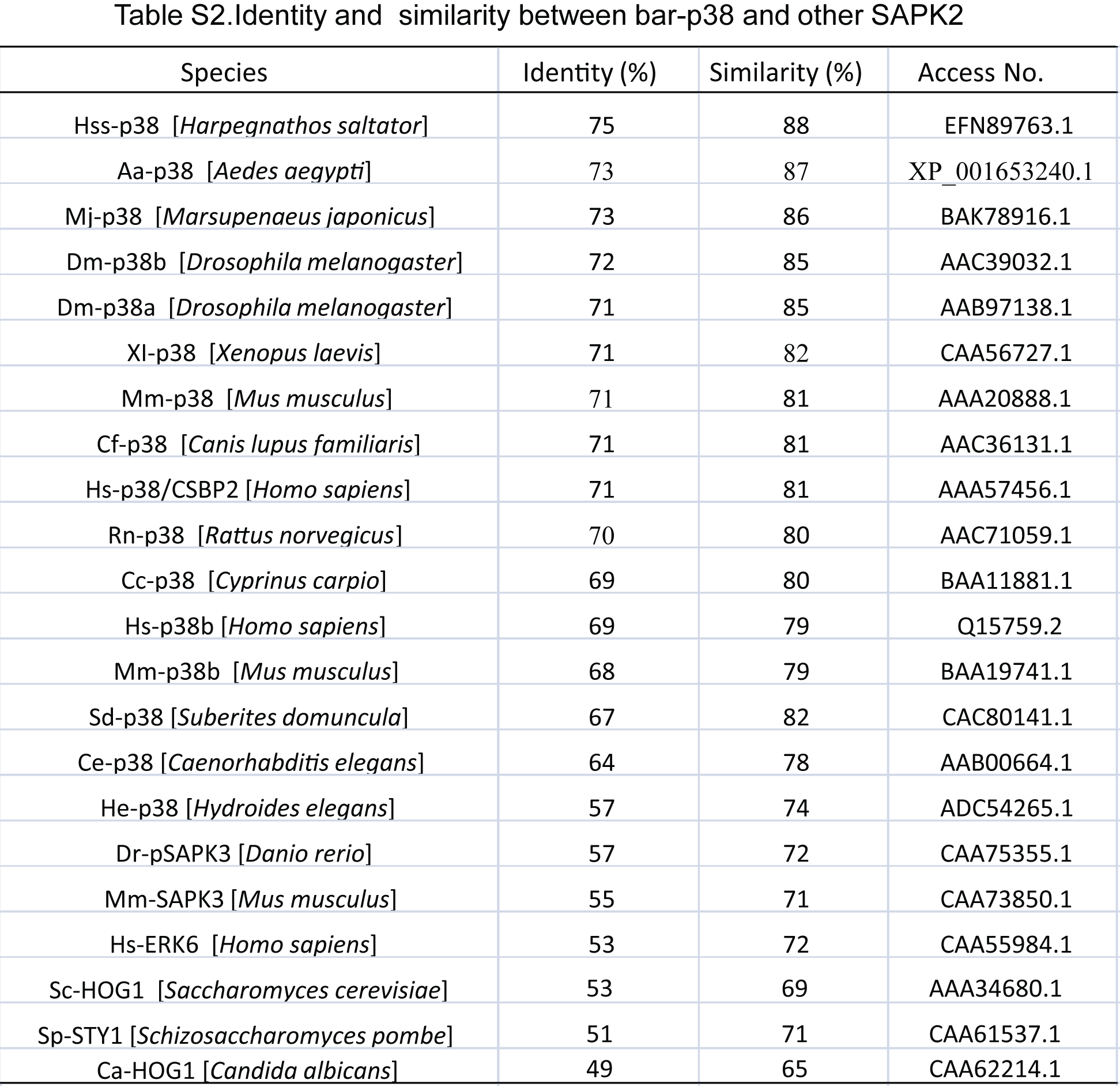

Supplement: Table S2 — Identity and similarity between bar-p38 and other SAPK2. (TIF) [file pone.0047195.s003.tif]
